# The cost-effectiveness of vaccinating against Lyme disease.

**DOI:** 10.3201/eid0505.990521

**Published:** 1999

**Authors:** D Prybylski


detected in digestive tracts of flies exposed to feces with oocysts. *C. parvum* oocysts were also numerous on maggot and pupa surfaces; approximately 150 and 320 oocysts were recovered per maggot and pupa, respectively.

Wild-caught flies belonged to the families *Calliphoridae* (96% of total flies), *Sarcophagidae* (2%), and *Muscidae* (2%). An average of eight flies was caught per trap, and more than 90% of flies harbored *C. parvum* oocysts. The number of trap-recovered *C. parvum* oocysts per fly was 2 to 246 (mean 73 oocysts per fly).

Synanthropic flies that breed in or come in contact with a fecal substrate contaminated with *C. parvum* oocysts can harbor these oocysts both externally and internally and will mechanically deposit them on other surfaces. Therefore, synanthropic flies can serve as mechanical vectors for *C. parvum* oocysts and under poor sanitary conditions could be involved in the transmission of human and animal cryptosporidiosis. The biology and ecology of synanthropic flies indicate that their potential for mechanical transmission of *C. parvum* oocysts can be high. The morphologic and AFS and IFA staining characteristics of *C. parvum* oocysts recovered from the exoskeletons of flies and identified in their fecal spots suggest that oocysts are still viable.

**Thaddeus K. Graczyk,*† Ronald Fayer,‡ Michael R. Cranfield,*† Barbara Mhangami-Ruwende,* Ronald Knight,* James M. Trout,§ and Heather Bixler§**

*Johns Hopkins University, Baltimore, Maryland, USA; †The Baltimore Zoo, Druid Hill Park, Baltimore, Maryland, USA; ‡U.S. Department of Agriculture, Beltsville, Maryland, USA; and §University of Pennsylvania, School of Veterinary Medicine, Philadelphia, Pennsylvania, USA

### References

1. Fayer R, Speer CA, Dubey JP. The general biology of *Cryptosporidium*. *Cryptosporidium* and cryptosporidiosis. In: Fayer R, editor. Boca Raton (FL): CRC Press; 1997. 1-42.
2. Graczyk TK, Fayer R, Cranfield MR. Zoonotic potential of cross-transmission of *Cryptosporidium parvum*: implications for waterborne cryptosporidiosis. Parasitol Today 1996;13:348-51.
3. Wallace GD. Experimental transmission of *Toxoplasma gondii* by filth-flies. Am J Trop Med Hyg 1971;20:411-3.
4. Hedges SA. Flies, gnats and midges. In: Malis A, editor. Handbook of pest control. Cleveland (OH): Franzak & Foster Co.; 1990. p. 621-84.
5. Greenberg B. Flies and diseases, biology and disease transmission. Princeton (NJ): Princeton University Press; 1973. p. 221.
6. Graczyk TK, Cranfield MR, Fayer R. Recovery of waterborne oocysts of *Cryptosporidium parvum* from water samples by the membrane-filter dissolution method. Parasitol Res 1997;83:121-5.
7. Graczyk TK, Cranfield MR, Fayer R. Evaluation of commercial enzyme immunoassay (EIA) and immunofluorescent antibody (IFA) test kits for detection of *Cryptosporidium* oocysts of species other than *Cryptosporidium parvum*. Am J Trop Med Hyg 1996;54:274-9.
8. Graczyk TK, Fayer R, Cranfield MR, Owens R. *Cryptosporidium parvum* oocysts recovered from water by the membrane filter dissolution method retain their infectivity. J Parasitol 1997;83:111-4.
9. Borror DJ, DeLong DM, Triplehorn CA. An introduction to the study of insects. Philadelphia (PA): Saunders College Publishing; 1981. p. 827.

### The Cost-Effectiveness of Vaccinating against Lyme Disease

**To the Editor:** The recent article by Meltzer and colleagues (1) is an important contribution to a pertinent public health issue: who should receive the newly licensed Lyme disease vaccine. Answering this question is a daunting task, given the scarcity of valid data. Estimates of the spectrum and prevalence of the long-term sequelae of Lyme disease remain controversial (2-4). In generating their cost-effectiveness model, Meltzer et al. examined the cost savings involved in preventing three categories of classic organ-specific Lyme disease sequelae (cardiovascular, neurologic, and arthritic); however, they did not take into account the potential cost savings from preventing cases of a generalized symptom complex known as post-Lyme syndrome, which includes persisting myalgia, arthralgia, headache, fatigue, and neurocognitive deficits. These generalized sequelae, which are recognized by the National Institutes of Health as late sequelae of Lyme disease, have been found to persist for years after antibiotic therapy (5,6). Two population-based retrospective cohort studies (7,8) among Lyme disease patients whose illness was diagnosed in the mid-1980s determined that one third to half had clinically corroborated post-Lyme syndrome symptoms years after the initial onset of disease. Although these studies were conducted 15 years ago, when optimal antibiotic regimen guidelines were still evolving, the estimated cost of averting these often-disabling nonorgan-specific symptoms should also be taken into account in estimated sensitivity analyses of vaccine cost-effectiveness. The cost of treating sequelae is weighted heavily in the cost-effectiveness models presented by Meltzer and colleagues, which adds importance to considering post-Lyme syndrome. Nevertheless, we recognize the difficulty of this modeling, especially in the absence of validated cost-of-treatment data for these generalized symptoms.

A point of correction is that Meltzer et al. erroneously cite one of these studies (7) to infer that the long-term clinical sequelae of Lyme disease lasted a mean of 6.2 years from the onset of disease. In this retrospective study, Shadick et al. evaluated 38 persons with a clinical history of Lyme disease a mean of 6.2 years from the onset of disease regardless of the presence of persisting symptoms; 25 of these patients had no residual symptoms at follow-up. To accurately estimate the duration of clinical sequelae, longitudinal evaluations of representative populations of Lyme disease patients will be required because late manifestations have been demonstrated months to years after diagnosis (9,10).

**Dimitri Prybylski**
University of Maryland School of Medicine,
Baltimore, Maryland, USA

### References

1. Meltzer MI, Dennis DT, Orloski KA. The cost-effectiveness of vaccinating against Lyme disease. Emerg Infect Dis 1999;5:321-8.
2. Ellenbogen C. Lyme disease. Shift in the paradigm? Arch Fam Med 1997;6:191-5.
3. Liegner KB. Lyme disease: the sensible pursuit of answers. J Clin Microbiol 1993;31:1961-3.
4. Sigal LH. Persisting symptoms of Lyme disease—possible explanations and implications for treatment [editorial]. J Rheumatol 1994;21:593-5.
5. National Institute of Allergy and Infectious Diseases. Research on chronic Lyme disease. National Institutes of Health Office of Communications Fact Sheet; May 1997.
6. National Institute of Allergy and Infectious Diseases. Emerging infectious diseases—NIAID research. National Institutes of Health Fact Sheet; Mar 1998.
7. Shadick NA, Phillips CB, Logigian EL, Steere AC, Kaplan RF, Berardi VP, et al. The long-term clinical outcomes of the disease. A population-based retrospective cohort study. Ann Intern Med 1994;121:560-7.
8. Asch ES, Bujak DI, Weiss M, Peterson MG, Weinstein A. Lyme disease: an infectious and post-infectious syndrome. J Rheumatol 1994;21:454-61.
9. Logigian EL, Kaplan RF, Steere AC. Chronic neurologic manifestations of Lyme disease. N Engl J Med 1990;323:1438-44.
10. Szer IS, Taylor E, Steere AC. The long-term course of Lyme arthritis in children. N Engl J Med 1991;325:159-63.

